# Effect of mucosal hydration on time-dependent mucoadhesion of polymeric nanocomposite microspheres to ovine nasal mucosa: relationship to swelling kinetics

**DOI:** 10.3389/fphar.2026.1974769

**Published:** 2026-10-14

**Authors:** Bissera Pilicheva, Plamen Zagorchev

**Affiliations:** 1 Department of Pharmaceutical Sciences and Social Pharmacy, Medical University Pleven, Pleven, Bulgaria; 2 Department of Biomedical Sciences, Medical University Pleven, Pleven, Bulgaria

**Keywords:** adhesion tension, drug delivery, hydration, mucoadhesion, nanocomposite polymeric microspheres, nasal mucosa, swelling kinetics

## Abstract

**Background:**

Mucoadhesion of hydrophilic polymeric carriers is strongly influenced by hydration, which promotes polymer-chain mobility and facilitates interactions with mucin. However, excessive hydration may compromise the mechanical integrity of the adhesive interface. This study investigated the temporal relationship between swelling kinetics and nasal mucoadhesion of polymeric nanocomposite microsphere systems under different mucosal hydration conditions.

**Methods:**

Two polymeric nanocomposite microsphere systems, IDB-MS and DFP-MS, were evaluated for swelling over 40 min using standardized compacted microsphere discs and for adhesion to excised ovine nasal mucosa under native/quasi-dry and highly hydrated conditions. Adhesion tension was determined over contact times ranging from 1 to 25 min. The effects of formulation and time on swelling and those of formulation, mucosal hydration, and contact time on adhesion tension were evaluated using factorial ANOVA.

**Results:**

Both systems showed comparable swelling kinetics, reaching their maximum experimentally observed swelling at 5 min (465.96% for IDB-MS and 470.00% for DFP-MS), with no significant main effect of formulation. Mucoadhesion was strongly affected by mucosal hydration and contact time, whereas formulation had no significant main effect. Adhesion tension was consistently higher under native/quasi-dry than under highly hydrated mucosal conditions. Spline interpolation estimated the time of maximum adhesion at approximately 4.0–4.5 min, whereas the highest experimentally measured adhesion tension occurred at the 5-min sampling point. However, the post-maximum decline in adhesion preceded the corresponding decrease in apparent swelling, particularly under highly hydrated conditions.

**Conclusion:**

Polymer swelling and mucoadhesion showed condition-dependent temporal correspondence, but swelling alone was insufficient to explain adhesive strength. The results support the hypothesis that mucoadhesion may depend on a balance between sufficient polymer hydration and preservation of interfacial cohesion; however, the present comparison of two hydration conditions does not establish an optimal hydration level. Mucosal hydration should therefore be considered an important experimental parameter in the evaluation of nasal mucoadhesive drug-delivery systems.

## 1 Introduction

Mucoadhesive drug delivery systems have attracted considerable interest as a strategy for prolonging the residence time of pharmaceutical formulations on mucosal surfaces and enhancing their interaction with the mucus layer. By establishing intimate contact with the mucus layer, mucoadhesive formulations may reduce clearance and prolong drug availability at the site of administration (Khutoryanskiy, 2011). This is particularly relevant to nasal drug delivery, where mucociliary clearance represents a major physiological barrier to prolonged drug–mucosa contact. Accordingly, mucoadhesive polymers have been investigated as a means of increasing nasal residence time and potentially improving local or systemic drug exposure. These potential benefits are particularly relevant to challenging therapeutic compounds, including peptides, proteins, and other macromolecular therapeutics with limited membrane permeability or stability (Chaturvedi et al., 2011; Djupesland, 2013). Recent studies further emphasize the role of mucoadhesive materials in intranasal delivery systems, while demonstrating that their performance is influenced by the physicochemical properties of both the polymer formulation and the nasal mucus environment (Kulkarni et al., 2023; Porfiryeva et al., 2026).

Mucoadhesion is a complex phenomenon that is generally explained by several complementary theories rather than by a single mechanism. The wetting theory emphasizes spreading and intimate contact between the polymer and mucosal surface, whereas the diffusion/interpenetration theory attributes adhesion to interpenetration and entanglement of polymer and mucin chains. The electronic theory considers the contribution of differences in electronic structure and charge distribution at the polymer–mucus interface, while the adsorption theory involves the formation of chemical and physical interactions, including hydrogen bonding, electrostatic interactions, van der Waals forces, and hydrophobic interactions. Fracture theory describes mucoadhesion in terms of the force or work required to separate the adhesive material from the mucus substrate (Smart, 2005; Smart, 2014). These mechanisms are not mutually exclusive and may operate simultaneously depending on polymer composition, molecular weight, functional groups, degree of crosslinking, mucus characteristics, and hydration state. Mucoadhesion is therefore a multifactorial phenomenon determined by the physicochemical properties of both the polymeric system and the mucus interface, rather than by a single material parameter (Khutoryanskiy, 2011; Schattling et al., 2017) Hydration should therefore not be regarded merely as an environmental condition, but as an integral component of the mucoadhesive process. The initial contact of the dry or partially hydrated polymer with the aqueous mucus environment promotes wetting and hydration, which subsequently facilitates polymer-chain mobility and the development of polymer–mucin interactions. Water therefore has a dual role: it facilitates spreading and molecular mobility, but excessive hydration can ultimately weaken the adhesive interface (Pathak, 2011; Agarwal and Aggarwal, 2015; Kulkarni et al., 2023).

In this context, swelling represents an important early event in the development of mucoadhesion, particularly for hydrophilic and swellable polymer systems (Smart, 2005; Andrews et al., 2009). Upon exposure to an aqueous environment, water penetrates the polymer matrix and may act as a plasticizing agent, reducing intermolecular interactions within the polymer network and increasing polymer-chain mobility (Andrews et al., 2009). This hydration-induced relaxation may increase the accessibility of functional groups capable of interacting with mucin and promote interpenetration between polymer chains and the mucus network. The expanded and hydrated polymer matrix may consequently increase the effective interfacial contact area and facilitate hydrogen bonding, electrostatic interactions, and other secondary interactions at the polymer–mucin interface (Hägerström and Edsman, 2001; Agarwal and Aggarwal, 2015). However, greater swelling should not be assumed to result directly in greater mucoadhesive strength. Both the extent and rate of swelling may influence the development of the adhesive interface. Limited hydration can lead to insufficient polymer-chain mobility and limited interpenetration, while excessive or very rapid hydration may result in dilution of the interfacial polymer phase and reduced cohesive strength of the hydrated layer. Therefore, the relationship between swelling and adhesion is likely to be dynamic and potentially nonlinear, because both insufficient and excessive hydration may be unfavorable for the development and maintenance of the adhesive interface. From this perspective, swelling kinetics can provide important information about the temporal evolution of mucoadhesive interactions, rather than simply describing a static physicochemical property of the formulation.

Despite the extensive literature on mucoadhesive polymers and the widespread use of swelling and mucoadhesion measurements in formulation development, these properties are commonly evaluated as separate characteristics. Although previous studies support the importance of hydration, swelling, and polymer-chain mobility in the establishment of polymer–mucin interactions, the temporal relationship between swelling and the emergence of measurable mucoadhesive strength appears to be less well characterized, particularly in polymeric microparticle systems intended for nasal administration. A kinetic approach that evaluates both phenomena over comparable time intervals may therefore provide additional insight into the relationship between polymer hydration and mucoadhesion beyond conventional endpoint measurements.

The two nanocomposite microsphere systems investigated in the present study were selected from previously developed formulations intended for intranasal drug delivery to the brain in the context of neurodegenerative disorders. One system contains idebenone (IDB), a lipophilic benzoquinone with antioxidant and mitochondrial electron-transfer properties, whereas the other contains deferiprone (DFP), a hydrophilic iron-chelating agent investigated in conditions associated with pathological iron accumulation and oxidative stress. Their pharmaceutical characteristics, therapeutic rationale, safety considerations, formulation development, and physicochemical properties have been described in detail in our previous studies (Boyuklieva et al., 2024a; 2024b). In the present work, IDB and DFP were not investigated for their pharmacological effects but served as components of two previously established nanocomposite microsphere systems used to examine hydration-dependent mucoadhesive behavior. The two compounds were not incorporated into a single formulation because of their markedly different physicochemical properties and the different formulation approaches required for their incorporation. Importantly, the present study was not designed to compare the pharmacological properties of IDB and DFP, but rather to determine whether the previously developed carrier systems exhibited differences in swelling and mucoadhesive behavior. Accordingly, the present study aimed to extend the previous characterization of IDB-MS and DFP-MS by investigating their time-dependent mucoadhesive behavior under highly hydrated mucosal conditions and comparing it with previously reported reference data obtained under native/quasi-dry conditions (Boyuklieva et al., 2024a; 2024b). In parallel, swelling kinetics of the two formulations were investigated to examine the temporal relationship between formulation hydration and mucoadhesive behavior. This approach was intended to determine how increased mucosal hydration modifies the time-dependent adhesion profile and to explore the relationship between swelling and mucoadhesion.

## 2 Materials and methods

### 2.1 Materials

Two previously developed polymeric nanocomposite microsphere formulations were investigated in the present study. Both formulations consisted of drug-loaded poly (ε-caprolactone) (PCL) nanoparticles incorporated into a sodium alginate matrix. The first formulation contained idebenone (IDB) and was designated IDB-MS, whereas the second contained deferiprone (DFP) and was designated DFP-MS. The development, preparation, and physicochemical characterization of IDB-MS and DFP-MS have been reported previously (Boyuklieva et al., 2024a; 2024b). In both formulations, drug-loaded PCL nanoparticles were incorporated into a sodium alginate matrix, followed by spray drying to obtain the nanocomposite microspheres. Detailed formulation compositions, preparation procedures, and physicochemical characterization have been reported previously (Boyuklieva et al., 2024a; 2024b).

### 2.2 Investigated nanocomposite microsphere formulations

The previously characterized systems were designated IDB-MS and DFP-MS and contained idebenone and deferiprone, respectively. Their previously determined mean particle diameters were 7.37 ± 2.40 and 6.05 ± 1.78 μm, respectively (Boyuklieva et al., 2024a; 2024b). The exact formulation compositions and preparation procedures were adopted from the respective published studies and were not modified in the present work. For detailed formulation composition, nanoparticle preparation, spray drying procedure, and physicochemical characterization, the original publications should be consulted (Boyuklieva et al., 2024a; 2024b).

### 2.3 Preparation of samples for the present kinetic study

Previously prepared IDB-MS and DFP-MS samples were used for the experiments. Prior to analysis, the samples were stored at 20 °C in desiccator until use. For each experiment, 30 mg of dry microspheres were compressed using a hydraulic press at 160 kPa to obtain standardized discs measuring 7 mm in diameter and 1 mm in height. The resulting discs were used for the swelling kinetics and mucoadhesion studies. The same initial sample mass was used for both formulations to standardize the samples used in the swelling and mucoadhesion experiments. Compaction was used to provide a reproducible sample geometry, constant initial mass, and defined contact area required for quantitative comparison of swelling and detachment-force measurements. The compacted discs therefore served as standardized experimental specimens of the microsphere formulations and were not intended to reproduce the physical state of freely dispersed microspheres following nasal administration.

### 2.4 Evaluation of mucoadhesive strength

The mucoadhesive ability of the nanocomposite microsphere formulations was evaluated by determining the maximum detachment force per unit area of freshly isolated ovine nasal mucosa, using an interface-force method previously applied to these systems (Boyuklieva et al., 2024b). Within 1 h after animal death, the nasal mucosa was excised and fixed immobile on a paraffin disc. PBS (pH 7.4) was used for storage and controlled moistening of the mucosal tissue. A standardized disc prepared by compression of 30 mg of microspheres as described in Section 2.3 was fixed onto a microscopic coverslip and brought into contact with the mucosal surface maintained at 30 °C ± 0.5 °C. Mucoadhesion was evaluated after contact times of 1, 3, 5, 10, 15, and 25 min under two experimental conditions: native/quasi-dry nasal mucosa, used without intentional prehydration or excess PBS exposure, and highly hydrated nasal mucosa, for which 100 µL of PBS was applied to the mucosal surface 30 s before contact with the microsphere disc. After the predefined contact time, physical separation was achieved by vertical displacement of the disc at a constant rate of 0.5 mm s^-1^. The maximum adhesive force generated during separation was measured using an interface system comprising an isometric force transducer TRI201 (LSI LETICA Scientific Instruments, Panlab S.L., Barcelona, Spain), a high-gain amplifier, and a filtering system. The amplifier output voltage was digitized using a microcontroller-operated 13-bit analog-to-digital converter. The maximum detachment force (Fmax) was normalized to the effective contact area (A) and expressed as adhesion tension (g/cm^2^; 1 g/cm^2^ = 98.07 Pa). For each formulation × mucosal hydration condition × contact-time combination, three independent experimental measurements were performed (n = 3), using a separate microsphere disc and a separate mucosal specimen for each measurement. Mucosal specimens used for the native/quasi-dry and highly hydrated experiments were obtained from the same mucosal tissue batch.

The mucoadhesion data obtained under the native/quasi-dry condition had been previously reported as part of the original characterization of the IDB-MS and DFP-MS formulations (Boyuklieva et al., 2024a; 2024b), whereas the corresponding data obtained under the highly hydrated condition have not been previously published. The native/quasi-dry and highly hydrated experiments were performed contemporaneously using the same mucosal tissue batch and under otherwise identical experimental conditions. In the present study, the previously reported native/quasi-dry data were replotted and reanalyzed as reference datasets for direct comparison with the contemporaneously obtained highly hydrated data and for the integrated analysis of mucoadhesion and swelling kinetics. The swelling data were generated specifically for the present study.

The ovine nasal mucosa used in this study was obtained as a by-product from a local abattoir. No animals were specifically bred, housed, euthanized, or sacrificed for the purposes of this study. Therefore, ethical approval for the use of the excised tissue was not required.

### 2.5 Evaluation of swelling kinetics

The bulk swelling behavior of the standardized compacted microsphere discs was evaluated in phosphate-buffered saline (PBS, pH 7.4) at 30 °C ± 0.5 °C as an aqueous environment relevant to the experimental conditions used for nasal mucoadhesion testing. A disc of microspheres of known mass (W_0_) was immersed in PBS (pH 7.4) at 30 °C ± 0.5 °C. Swelling measurements were performed at 1, 3, 5, 10, 15, 25 and 40 min. At each predetermined time point, the hydrated samples were removed from the medium and gently blotted to remove excess surface liquid prior to weighing. The same blotting procedure was applied consistently to all samples and time points to minimize handling-related variability. The swollen mass at time *t* was designated W_t_, whereas the initial dry mass was designated W_0_. The swelling index (SI) was calculated according to:

$$\text{SI }\left(\%\right)=\frac{W_{t}‐W_{0}}{W_{0}}\times 100$$

The resulting swelling profiles were expressed as swelling index as a function of hydration time. Measurements were performed in triplicate (n = 3) for each formulation and time point. Swelling was evaluated at time points corresponding to those used in the mucoadhesion experiments, with an additional measurement at 40 min.

### 2.6 Data processing and statistical analysis

All quantitative data were processed using the individual experimental measurements obtained for each formulation, hydration condition, and contact time. Data are presented as mean ± SD of three independent experimental measurements (n = 3) for each formulation × mucosal hydration condition × contact-time combination. For the mucoadhesion experiments, the maximum detachment force recorded during separation of the compacted microsphere disc from the nasal mucosa was normalized to the effective contact area and expressed as adhesion tension. Time-dependent mucoadhesive profiles were further characterized by the time to maximum adhesion tension (t_max_), time to reach 50% and 75% of maximum adhesion during the ascending phase (t_50up_ and t_75up_, respectively), time to decrease to 75% of maximum adhesion during the descending phase (t_75down_), and time to reach a stable late-phase adhesion level (t_stable_). For descriptive characterization of the temporal profiles, cubic spline interpolation was applied to the mean adhesion-tension values obtained at the experimentally investigated contact times (SRS1 software, LLC). The interpolation was used to estimate characteristic temporal parameters between the discrete experimental time points, including tmax, t50up, t75up, t75down, and tstable. The cubic spline consisted of piecewise third-order polynomial functions joined at the experimental time points with continuity of the function and its first and second derivatives. The interpolation was used as a descriptive numerical procedure and was not intended to represent a mechanistic or parametric kinetic model. Accordingly, spline-derived temporal parameters represent interpolated estimates between experimentally sampled time points rather than directly observed experimental values. The highest experimentally measured adhesion tension occurred at the 5-min sampling point under all experimental conditions. The duration for which adhesion tension remained above 75% of its maximum value was calculated as:

$$\Delta t_{>75\%}=t_{75\text{down}}\;–\;t_{75\text{up}}$$

For the swelling experiments, the swelling index (SI) was calculated from the initial dry mass and the mass of the hydrated sample. The swelling profile was characterized by the maximum swelling index (SI_max_), time to maximum swelling (t_SImax_), apparent swelling rates over selected time intervals, and the relative decrease in SI after SI_max_. The integrated swelling response over 40 min was expressed as AUC_0–40_. AUC_0_–_40_ was calculated using the trapezoidal rule. The effects of formulation and time on swelling index were evaluated using two-way factorial analysis of variance (ANOVA), including the formulation × time interaction. The effects of formulation, mucosal hydration condition, and contact time on adhesion tension were evaluated using three-way factorial ANOVA, including all two-way interactions and the three-way interaction. Contact time was treated as a categorical factor. When statistically significant main effects or interactions were identified, *post hoc* pairwise comparisons were performed with Holm adjustment for multiple comparisons. Effect sizes were expressed as partial eta squared (ηp^2^). Statistical significance was set at p < 0.05. Statistical analyses were performed using GraphPad Prism (v. 6.01, GraphPad Software Inc.).

## 3 Results

### 3.1 Mucoadhesive performance of the nanocomposite microspheres

Both nanocomposite microsphere formulations, IDB-MS and DFP-MS, exhibited mucoadhesive activity toward excised ovine nasal mucosa, with adhesion already detectable after 1 min of contact. Adhesion tension increased during the initial contact period, reached its highest experimentally measured value at 5 min, and decreased thereafter toward a lower, relatively stable late-phase level. No statistically significant differences in adhesion tension were observed between IDB-MS and DFP-MS when compared under the same experimental conditions. Accordingly, the two formulations showed similar overall time-dependent mucoadhesive profiles (Figure 1).

**FIGURE 1:**
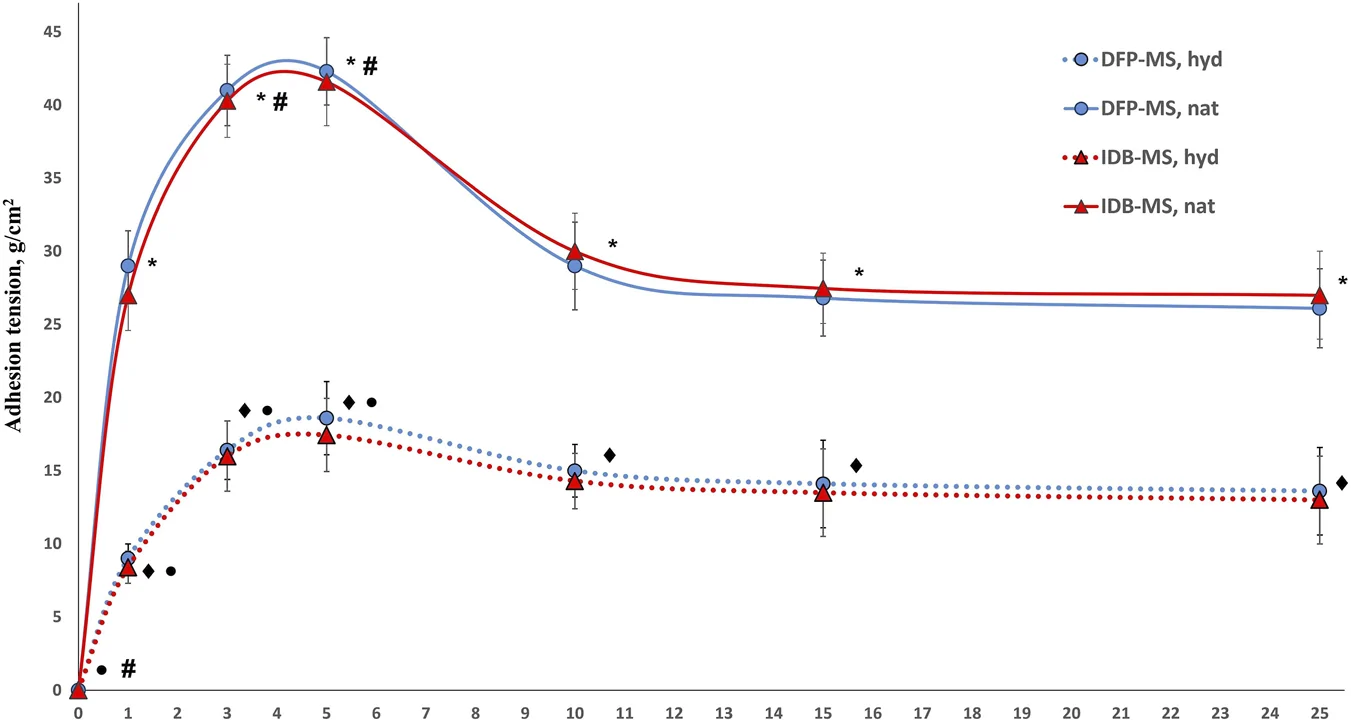
Time-dependent adhesion tension of IDB-MS and DFP-MS on native/quasi-dry (IDB-MS, nat; DFP-MS, nat) and highly hydrated (IDB-MS, hyd; DFP-MS, hyd) nasal mucosa. Data are presented as mean ± SD (n = 3). The symbols *, #, **●** and **♦** indicate statistically significant differences compared with the initial time point (* and **♦**) or the 15-min value corresponding to the steady-state phase (# and **●**). The native/quasi-dry data were previously reported during the original characterization of IDB-MS and DFP-MS (Boyuklieva et al., 2024a; 2024b) and are replotted here for direct comparison with the contemporaneously obtained, previously unpublished highly hydrated-condition data. Both hydration conditions were evaluated using the same mucosal tissue batch.

### 3.2 Effect of formulation, mucosal hydration, and contact time on mucoadhesive strength

Adhesion tension was significantly affected by mucosal hydration and contact time, whereas no significant main effect of formulation was detected (Table 1). Three-way factorial ANOVA revealed a pronounced main effect of mucosal hydration on adhesion tension (F (1,48) = 954.24, p < 0.0001, ηp^2^ = 0.952). Adhesion tension was consistently higher under native/quasi-dry conditions than under highly hydrated conditions. At 5 min, adhesion tension under native/quasi-dry conditions was approximately 2.3-fold higher than that observed under highly hydrated conditions. The magnitude of this effect was particularly evident during the early and maximum phases of mucoadhesion. At 5 min, the highest experimentally measured adhesion tensions were 42.3 ± 2.3 g/cm^2^ and 41.6 ± 3.0 g/cm^2^ for the two formulations under native/quasi-dry conditions, compared with 18.6 ± 2.5 g/cm^2^ and 17.45 ± 2.5 g/cm^2^, respectively, under highly hydrated conditions. Contact time also exerted a significant effect on adhesion tension (F (5,48) = 44.77, p < 0.0001, ηp^2^ = 0.823). Under all experimental conditions, adhesion tension increased during the initial contact period, reached its highest measured value at 5 min, and subsequently decreased toward a relatively stable level at longer contact times. Importantly, a significant hydration × time interaction was observed (F (5,48) = 12.74, p < 0.0001, ηp^2^ = 0.570), demonstrating that the effect of mucosal hydration on adhesion tension varied with contact time. In contrast, microparticle formulation had no significant main effect on adhesion tension (F (1,48) = 0.48, p = 0.493, ηp^2^ = 0.010). No significant formulation × hydration (F (1,48) = 0.21, p = 0.651), formulation × time (F (5,48) = 0.18, p = 0.968), or formulation × hydration × time interaction (F (5,48) = 0.17, p = 0.971) was detected. Overall, mucosal hydration and contact time accounted for the major observed variation in adhesion tension, whereas the two formulations exhibited comparable mucoadhesive performance under equivalent experimental conditions.

**TABLE 1:** Effect of formulation, mucosal hydration, and contact time on adhesion tension analysed by three-way factorial ANOVA.

Effect	F	df	p	Partial η^2^
Formulation	0.48	1,48	0.493	0.010
Hydration	**954.24**	1,48	**<0.0001**	**0.952**
Contact time	**44.77**	5,48	**<0.0001**	**0.823**
Formulation × hydration	0.21	1,48	0.651	0.004
Formulation × time	0.18	5,48	0.968	0.019
Hydration × time	**12.74**	5,48	**<0.0001**	**0.570**
Formulation × hydration × time	0.17	5,48	0.971	0.018

Bold formatting highlights the factors and interaction terms of particular interest in the interpretation of the results.

### 3.3 Post hoc analysis of the hydration × contact time interaction

Because a significant hydration × contact time interaction was identified, simple-effects analyses were performed to determine the effect of mucosal hydration at each contact time and to characterize the time-dependent changes within each hydration condition. Pairwise comparisons were based on the pooled residual variance from the factorial ANOVA, and p-values were adjusted for multiple comparisons using the Holm procedure.

#### 3.3.1 Effect of mucosal hydration at individual contact times

Adhesion tension was significantly higher under native/quasi-dry conditions at every contact time (all Holm-adjusted p < 0.0001; Table 2). The largest relative difference was observed at 1 min (3.22-fold), whereas the native/quasi-dry-to-hydrated ratio decreased to approximately 2-fold at 10–25 min.

**TABLE 2:** Simple effects of mucosal hydration on adhesion tension at individual contact times. Values represent estimated marginal means averaged across the two microsphere formulations.

Time, min	Native/quasi-dry, g/cm^2^	Highly hydrated, g/cm^2^	Mean difference, g/cm^2^	Native/hydrated ratio	95% CI of difference, g/cm^2^	Adjusted p
1	28.00	8.70	19.30	3.22	16.40–22.20	<0.0001
3	40.65	16.20	24.45	2.51	21.55–27.35	<0.0001
5	41.95	18.03	23.93	2.33	21.03–26.82	<0.0001
10	29.50	14.65	14.85	2.01	11.95–17.75	<0.0001
15	27.14	13.80	13.34	1.97	10.44–16.23	<0.0001
25	26.55	13.30	13.25	2.00	10.35–16.15	<0.0001

#### 3.3.2 Time-dependent changes under highly hydrated conditions

Under highly hydrated conditions, adhesion tension increased markedly during the initial phase. The estimated marginal mean increased from 8.70 g/cm^2^ at 1 min to 16.20 g/cm^2^ at 3 min and reached its highest measured value of 18.03 g/cm^2^ at 5 min. Compared with the 5-min maximum, adhesion tension was significantly lower at 1 min (adjusted p < 0.0001). The difference between 3 and 5 min was not statistically significant (adjusted p = 0.212). After the maximum, adhesion tension decreased to 14.65 g/cm^2^ at 10 min, 13.80 g/cm^2^ at 15 min, and 13.30 g/cm^2^ at 25 min. The 5-min value was significantly higher than those at 10, 15, and 25 min, whereas the 3-min value did not differ significantly from either the 5-min value or the later time points after Holm correction. When all pairwise comparisons among contact times were considered within the highly hydrated condition, no statistically significant differences were detected among 3, 10, 15, and 25 min after correction for multiple comparisons, whereas the 1-min value remained significantly lower than all later time points.

#### 3.3.3 Time-dependent changes under native/quasi-dry conditions

Under native/quasi-dry conditions, adhesion tension increased from 28.00 g/cm^2^ at 1 min to 40.65 g/cm^2^ at 3 min and reached its highest measured value of 41.95 g/cm^2^ at 5 min. The 5-min value was significantly higher than that observed at 1 min (adjusted p < 0.0001). In contrast, no statistically significant difference was detected between the 3- and 5-min values (adjusted p = 0.372). After 5 min, adhesion tension decreased markedly. Values at 10, 15, and 25 min were all significantly lower than the 5-min maximum (all adjusted p < 0.0001). No statistically significant differences were detected among the late-phase values at 10, 15, and 25 min after correction for multiple comparisons. The 1-min value also did not differ significantly from the values measured at 10, 15, or 25 min.

The profile can therefore be descriptively divided into three phases: an initial increase between 1 and 3 min, a high-adhesion phase between 3 and 5 min, and a lower late-phase level from approximately 10 min onward.

### 3.4 Hydration-dependent kinetics of mucoadhesion

Although both hydration conditions produced a similar overall pattern of adhesion development, spline-derived kinetic parameters differed between the two mucosal hydration conditions (Table 3). The estimated time to maximum adhesion tension (t_max_) fell within a relatively narrow interval under all experimental conditions, ranging from 4.0 to 4.2 min under highly hydrated conditions and from 4.2 to 4.5 min under native/quasi-dry conditions. The interpolated profiles indicated a faster ascending phase under highly hydrated conditions. The time required to reach 50% of maximum adhesion tension was 0.5–0.6 min for highly hydrated mucosa compared with 1.0–1.1 min for native/quasi-dry mucosa. A more pronounced difference was observed for the time required to reach 75% of maximum adhesion tension, which was 1.1–1.2 min under highly hydrated conditions and 2.5–2.6 min under native/quasi-dry conditions. Differences were also observed during the descending phase. Adhesion tension decreased to 75% of its maximum at approximately 7.2 min under highly hydrated conditions, whereas the corresponding time was approximately 10.9–11.0 min under native/quasi-dry conditions. Consequently, the calculated duration during which adhesion tension remained above 75% of its maximum value (Δt_>75%)_ was 6.0–6.1 min under highly hydrated conditions and 8.3–8.5 min under native/quasi-dry conditions. The stable adhesion level was reached at approximately 15 min under highly hydrated conditions and at approximately 11 min under native/quasi-dry conditions.

**TABLE 3:** Derived characteristic kinetic parameters of adhesion tension.

Parameter	IDB-MS, hydrated	IDB-MS, native	DFP-MS, hydrated	DFP-MS, native
t_max_, min	4.0	4.2	4.2	4.5
t_50up_, min	0.6	1.1	0.5	1.0
t_75up_, min	1.2	2.6	1.1	2.5
t_75down_, min	7.2	10.9	7.2	11.0
t_stable_, min	15.0	11.0	15.0	11.0
Δt_>75%,_ min	6.0	8.3	6.1	8.5

### 3.5 Swelling kinetics

Gravimetric swelling of the compacted IDB-MS and DFP-MS discs was evaluated over 40 min (Figure 2). For IDB-MS, the swelling index increased from 217.45% ± 6.90% at 1 min to 319.97% ± 12.90% at 3 min and reached its highest experimentally observed SI of 465.96% ± 6.90% at 5 min. A comparable swelling level was maintained at 10 min (460.47% ± 13.50%), after which the swelling index progressively decreased to 404.17% ± 14.50% at 15 min, 395.49% ± 11.80% at 25 min, and 378.42% ± 5.10% at 40 min. A closely comparable swelling profile was observed for DFP-MS. The swelling index increased from 203.44% ± 4.92% at 1 min to 310.93% ± 2.87% at 3 min and reached its highest experimentally observed SI of 470.00% ± 8.96% at 5 min. The swelling index remained high at 10 min (455.52% ± 7.26%) and subsequently decreased to 416.02% ± 15.33% at 15 min, 392.49% ± 11.83% at 25 min, and 370.42% ± 5.10% at 40 min. Descriptively, both profiles comprised three phases: a rapid increase up to 5 min, maintenance of near-maximum swelling at 10 min, and a progressive post-maximum decrease thereafter. For IDB-MS, the swelling index subsequently decreased by 13.26% relative to the experimentally observed SImax at 15 min, 15.12% at 25 min, and 18.79% at 40 min. For DFP-MS, the corresponding decreases were 11.48%, 16.49%, and 21.19%, respectively. Two-way factorial ANOVA demonstrated a highly significant effect of time on the swelling index (F (6,28) = 482.40, p < 0.0001). In contrast, the main effect of formulation was not statistically significant (F (1,28) = 1.16, p = 0.291). The formulation × time interaction was also not statistically significant (F (6,28) = 1.15, p = 0.359), providing no evidence of a formulation-dependent difference in the time course of swelling.

**FIGURE 2:**
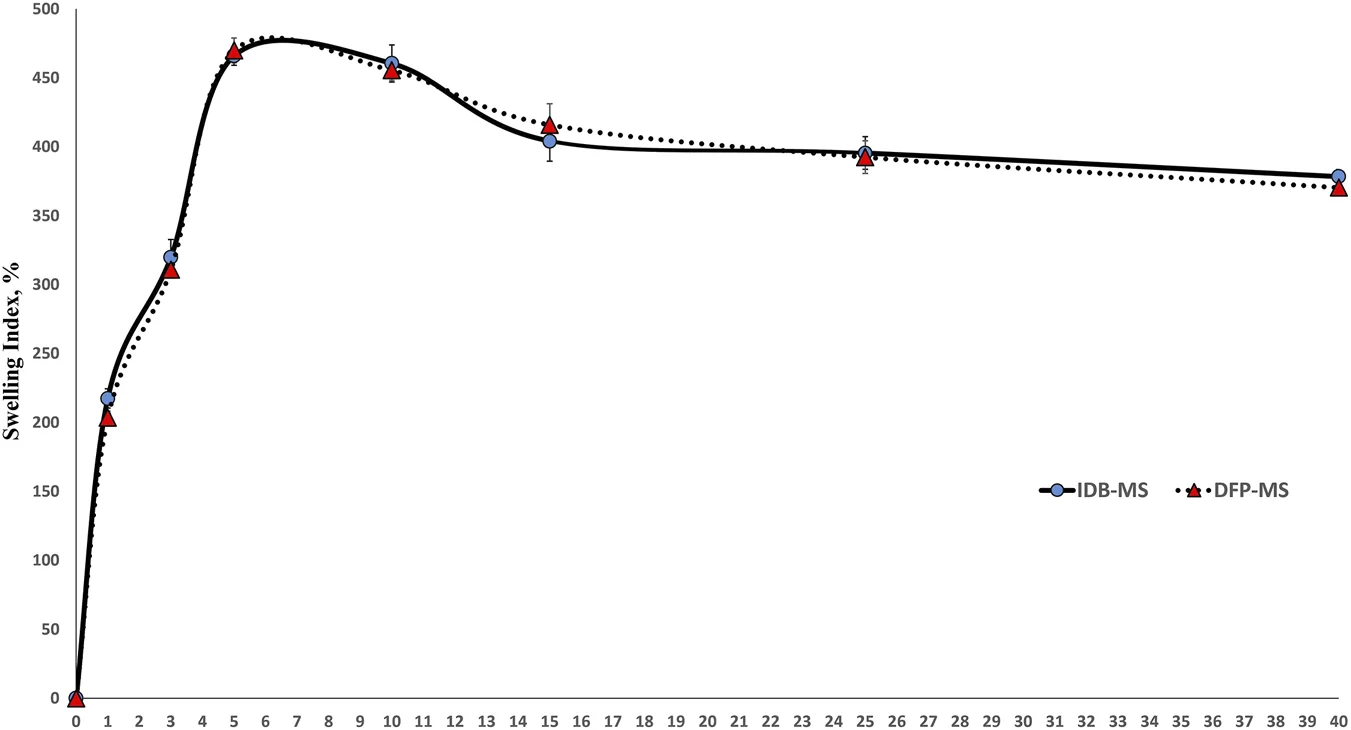
Time-dependent gravimetric swelling profiles of standardized compacted IDB-MS and DFP-MS microsphere discs in phosphate-buffered saline (PBS, pH 7.4) at 30 °C ± 0.5 °C. Data are presented as mean ± SD (n = 3 independent experimental measurements per formulation and time point). The effects of formulation, hydration time, and their interaction were evaluated using two-way factorial ANOVA, followed by Holm-adjusted *post hoc* comparisons where appropriate. A significant effect of time was observed (p < 0.0001), whereas neither the main effect of formulation (p = 0.291) nor the formulation × time interaction (p = 0.359) was statistically significant.

The derived swelling parameters further demonstrated the close similarity between the two microparticle systems (Table 4). The maximum swelling index was 465.96% for IDB-MS and 470.00% for DFP-MS, with both systems reaching their maximum at 5 min. The estimated times to reach 50% and 75% of maximum swelling were 1.30 and 3.40 min for IDB-MS and 1.59 and 3.52 min for DFP-MS, respectively. The integrated swelling response over 40 min was also highly comparable, with AUC_0–40_ values of 15,712.38 and 15,654.03%·min for IDB-MS and DFP-MS, respectively.

**TABLE 4:** Derived kinetic parameters of gravimetric swelling.

Kinetic parameter	IDB-MS	DFP-MS
SI_max_, %	465.96	470.00
t_SImax_, min	5.00	5.00
t_50% max_, min	1.30	1.59
t_75% max_, min	3.40	3.52
Swelling rate, 1–3 min, % min^-1^	51.26	53.75
Swelling rate, 3–5 min, % min^-1^	72.99	79.53
Decrease from SI_max_ at 40 min, %	18.79	21.19
AUC_0–40_, % min	15,712	15,654

### 3.6 Temporal correspondence between swelling and mucoadhesion

Comparison of the time-dependent swelling and mucoadhesion profiles showed temporal correspondence between gravimetric swelling and the development of mucoadhesive strength (Table 5). The closest temporal correspondence between the two processes was observed for the time to maximum response: experimentally observed SI_max_ occurred at 5 min, whereas spline-derived estimate of tmax for adhesion ranged from 4.0 to 4.5 min. The ascending phases differed according to mucosal hydration, with adhesion developing earlier than bulk swelling under highly hydrated conditions and showing closer temporal correspondence with swelling under native/quasi-dry conditions. A comparable time scale was observed for the development of mucoadhesion. Under highly hydrated mucosal conditions, 50% of maximum adhesion was reached at 0.5–0.6 min and 75% at 1.1–1.2 min, with maximum adhesion occurring at 4.0–4.2 min. Under native/quasi-dry conditions, the corresponding times were 1.0–1.1 min for 50% of maximum adhesion and 2.5–2.6 min for 75%, while maximum adhesion was reached at 4.2–4.5 min. Thus, maximum swelling and maximum mucoadhesive strength occurred within the same narrow time interval. The estimated adhesion maxima occurred 0.5–1.0 min before the experimentally observed swelling maximum. The temporal correspondence was particularly evident under native/quasi-dry mucosal conditions. The estimated t_75_ values for swelling were 3.40 and 3.52 min for IDB-MS and DFP-MS, respectively, compared with t_75_ values for adhesion of 2.5–2.6 min. Under highly hydrated mucosal conditions, however, 75% of maximum adhesion was reached substantially earlier (1.1–1.2 min) than 75% of maximum swelling. Following the maximum, both processes exhibited a declining phase. The swelling index remained close to its maximum between 5 and 10 min and subsequently decreased progressively up to 40 min. In parallel, adhesion tension decreased after reaching its maximum at approximately 4–5 min. The adhesion tension declined to 75% of its maximum at approximately 7.2 min under highly hydrated conditions and at 10.9–11.0 min under native/quasi-dry conditions. Taken together, the kinetic profiles demonstrate temporal correspondence between the development of polymer swelling and mucoadhesive strength, particularly with respect to the occurrence of their maxima within the first 5 min of contact.

**TABLE 5:** Direct comparison of characteristic temporal parameters of swelling and mucoadhesion under different mucosal hydration conditions.

Parameter	IDB-MS swelling	DFP-MS swelling	Adhesion, hydrated mucosa	Adhesion, native/quasi-dry mucosa
t_50_, min	1.30	1.59	0.5–0.6	1.0–1.1
t_75_, min	3.40	3.52	1.1–1.2	2.5–2.6
t_max_, min	5.00	5.00	4.0–4.2	4.2–4.5
t_75down_, min	—*	—*	7.2	10.9–11.0

*The swelling index remained above 75% of SI_max_ throughout the 40-min observation period.

Under highly hydrated mucosal conditions, the development of mucoadhesion preceded the corresponding swelling response. Adhesion increased rapidly during the initial contact period, reaching 50% and 75% of its maximum within approximately 0.5–0.6 and 1.1–1.2 min, respectively. In comparison, the corresponding swelling levels were reached later, with t_50_ values of 1.30 and 1.59 min and t_75_ values of 3.40 and 3.52 min for IDB-MS and DFP-MS, respectively. Despite these differences during the ascending phase, the maxima of the two processes occurred within a relatively narrow time interval. Maximum adhesion was reached at approximately 4.0–4.2 min, whereas the maximum swelling index was observed at 5 min for both microparticle systems. Following the maximum, the normalized adhesion decreased more rapidly than the swelling index. Adhesion tension declined to 75% of its maximum at approximately 7.2 min, whereas both microparticle systems retained more than 75% of their maximum swelling throughout the entire 40-min observation period. At 40 min, IDB-MS and DFP-MS retained approximately 81.2% and 78.8% of their respective maximum swelling indices. Thus, under highly hydrated conditions, the early development of mucoadhesion was faster than the swelling response, while the post-maximum decrease in adhesion occurred considerably earlier than the corresponding decrease in swelling.

Under native/quasi-dry mucosal conditions, the temporal development of mucoadhesion showed a closer correspondence with the swelling kinetics. Adhesion reached 50% of its maximum at approximately 1.0–1.1 min and 75% at 2.5–2.6 min. The corresponding t_50_ values for swelling were 1.30 and 1.59 min for IDB-MS and DFP-MS, while t_75_ values were 3.40 and 3.52 min, respectively. Maximum adhesion was reached at approximately 4.2–4.5 min, closely preceding the experimentally observed maximum swelling at 5 min. Thus, both the ascending phase and the time to maximum showed close temporal correspondence between the development of mucoadhesive strength and polymer swelling under native/quasi-dry conditions. After reaching their respective maxima, both processes entered a declining phase, although the decrease in adhesion remained faster than the decrease in swelling. Adhesion tension decreased to 75% of its maximum at approximately 10.9–11.0 min, whereas the swelling index remained above 75% of SI_max_ throughout the 40-min observation period. Compared with the highly hydrated condition, the native/quasi-dry mucosa therefore showed a closer temporal correspondence between the ascending phases of swelling and mucoadhesion and a longer persistence of high relative adhesion after the maximum.

Because swelling index and adhesion tension are expressed in different physical units and differ substantially in absolute magnitude, direct comparison of the original profiles does not readily reveal differences in their relative temporal development. Therefore, the swelling and adhesion data were additionally normalized to the respective maximum value of each experimental series. This normalization was used solely to compare the relative timing and shape of the kinetic profiles and does not represent an additional independent dataset or statistical analysis. The normalized profiles further demonstrated that the relationship between swelling and mucoadhesion depended on the hydration state of the mucosal surface (Figures 3A,B). Under highly hydrated conditions, the adhesion response developed considerably faster than swelling during the initial phase, but also decreased earlier after reaching its maximum. In contrast, under native/quasi-dry conditions, the ascending adhesion profile more closely followed the development of swelling, with t_50_, t_75_, and tmax occurring within relatively similar time ranges. The time to maximum response showed the closest correspondence: maximum adhesion was reached at 4.0–4.2 min under highly hydrated conditions and at 4.2–4.5 min under native/quasi-dry conditions, compared with a t_SImax_ of 5 min for both microparticle systems. After the maximum, however, adhesion decreased considerably faster than swelling under both hydration conditions.

**FIGURE 3:**
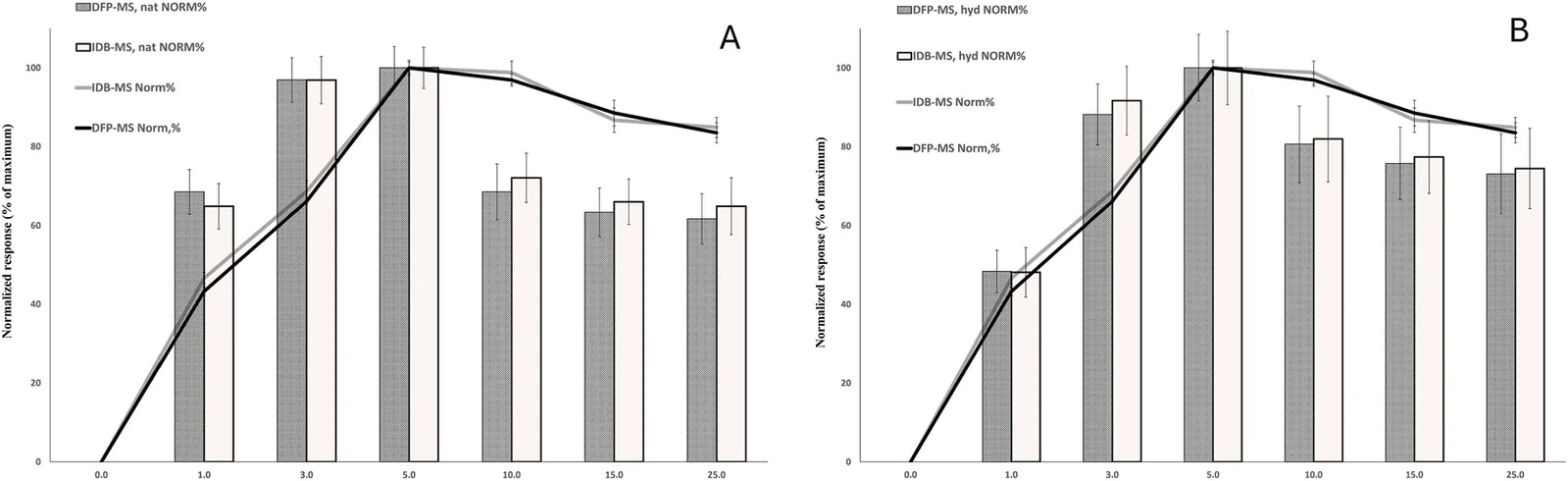
Temporal correspondence between swelling and adhesion tension of the investigated microsphere systems under different mucosal hydration conditions. Time-dependent swelling and adhesion profiles were normalized to the maximum value of the corresponding experimental series and expressed as percentage of the respective maximum response. Normalization was performed to facilitate comparison of the relative temporal profiles of swelling and adhesion, which are expressed in different physical units, and does not represent an independent experimental dataset. **(A)** Adhesion tension (bars) under native/quasi-dry nasal mucosa compared with the corresponding swelling profiles of IDB-MS and DFP-MS (curves). **(B)** Adhesion tension (bars) under highly hydrated nasal mucosa compared with the swelling profiles (curves). Under highly hydrated conditions, the development of adhesion preceded the swelling response, whereas under native/quasi-dry conditions, the ascending phases of swelling and mucoadhesion showed closer temporal correspondence. Data are presented as mean ± SD (n = 3 independent experimental measurements per formulation and time point). Error bars represent standard deviations.

## 4 Discussion

### 4.1 General mucoadhesive behavior of the microparticle systems

Both investigated nanocomposite microsphere systems exhibited measurable mucoadhesion to ovine nasal mucosa, with adhesion tension increasing during the initial contact period and subsequently declining toward a lower late-phase level. Importantly, no significant formulation-dependent effect was detected, whereas mucosal hydration and contact time exerted significant effects on adhesion tension. The present study was not designed to determine the contribution of individual formulation components to mucoadhesion. Both IDB-MS and DFP-MS were previously developed nanocomposite microsphere systems and were investigated here to determine whether the effects of mucosal hydration and contact time were reproducible across two related formulations. Accordingly, the absence of an unloaded control formulation and the lack of systematic variation in alginate content do not permit conclusions regarding the specific contribution of the drug payload, nanoparticle phase, or alginate matrix to the observed mucoadhesive behavior. Such formulation-dependent effects would require a dedicated experimental design including appropriate unloaded controls and systematic variation of polymer composition. The absence of a significant formulation effect was accompanied by highly similar swelling profiles of IDB-MS and DFP-MS, which reached nearly identical maximum swelling indices. The observed time-dependent profile is consistent with models in which initial contact and wetting are followed by progressive consolidation of the adhesive interface, involving hydration-dependent polymer-chain mobility, interpenetration of polymer and mucin chains, and formation of secondary interactions. Smart (2005) described contact and consolidation as the two principal stages of mucoadhesion, emphasizing that their relative contribution depends on the properties of both the formulation and the biological interface. This framework is particularly relevant to hydrophilic polymeric systems. Hydration promotes polymer swelling and increases chain mobility, thereby facilitating intimate contact with mucus and enabling interpenetration and secondary interactions with mucin. However, the relationship between hydration and adhesion is not necessarily monotonic, since insufficient hydration may restrict polymer-chain mobility whereas excessive hydration may weaken the adhesive interface.

### 4.2 Mucosal hydration as a major determinant of mucoadhesive strength

The most prominent finding of the present study was the strong dependence of adhesion tension on mucosal hydration. The factorial analysis identified mucosal hydration as the factor associated with the largest effect size on adhesion tension. At all investigated contact times, adhesion tension was substantially higher on native/quasi-dry mucosa than on highly hydrated mucosa. The magnitude of this difference was greatest during the early stage of interaction: the native-to-hydrated adhesion ratio was approximately 3.2 at 1 min, decreased to approximately 2.5 at 3 min and 2.3 at 5 min, and subsequently approached approximately two at longer contact times. The significant hydration × time interaction further demonstrates that hydration did not simply produce a constant reduction in adhesion strength but modified the temporal development of the adhesive interaction. The lower adhesion observed under highly hydrated conditions may initially appear counterintuitive because hydration is required for activation of hydrophilic mucoadhesive polymers. However, the literature clearly distinguishes between sufficient hydration, which promotes polymer-chain mobility and mucoadhesive interactions, and excessive hydration, which may weaken the adhesive interface through dilution of the interfacial polymer phase and reduced cohesive strength. Hydration promotes polymer-chain mobility and interaction with the mucus network, whereas excessive water may dilute the polymer at the interface and reduce the density and mechanical strength of polymer–mucin interactions. This is consistent with the established concept of an optimal hydration window for mucoadhesion, beyond which overhydration may weaken the adhesive joint (Ugwoke et al., 2001; Smart, 2005; Boddupalli et al., 2010; Khutoryanskiy, 2011). Thus, the substantially lower adhesion observed under highly hydrated conditions demonstrates that increasing water availability does not necessarily increase mucoadhesive strength and is consistent with the hypothesis that excessive hydration may weaken the adhesive interface.

### 4.3 Swelling kinetics and development of the mucoadhesive interface

Both IDB-MS and DFP-MS demonstrated rapid and extensive swelling. Their swelling indices increased steeply during the first 5 min and reached essentially identical maxima of approximately 466%–470%. The absence of a significant formulation effect and formulation × time interaction indicates that the two systems had comparable swelling kinetics. Swelling is potentially relevant to the establishment of the mucoadhesive interface. Hydration can increase polymer-chain mobility and may enhance the accessibility of functional groups involved in interactions with mucus. The diffusion theory of mucoadhesion proposes that sufficiently mobile polymer chains penetrate into the mucin network, with the extent of interpenetration depending on polymer-chain flexibility, molecular structure, contact time and hydration (Boddupalli et al., 2010; Khutoryanskiy, 2011). This mechanism is also relevant to alginate-containing systems. Alginate possesses abundant hydroxyl and carboxyl groups and has well-established swelling and mucoadhesive properties. Its interaction with mucin has been attributed primarily to non-covalent interactions, including hydrogen bonding, while hydration and relaxation of polymer chains facilitate intimate contact with the mucus layer. Molecular-scale AFM measurements have also directly demonstrated adhesion between mucin and calcium-alginate gels and have shown that the work of adhesion increases with contact time (Szekalska et al., 2018). Importantly, experimental nasal microparticle studies support the coexistence of swelling and mucoadhesion in alginate-based carriers. Gavini et al. (2005) reported that spray-dried alginate/chitosan microparticles intended for nasal administration showed both swelling and good *in vitro* mucoadhesive properties and underwent swelling and gel formation during *ex vivo* experiments with ovine nasal mucosa. Alginate microspheres have also been investigated specifically as mucoadhesive nasal carriers, with *ex vivo* studies on ovine nasal mucosa demonstrating substantial mucoadhesion (Szekalska et al., 2023a).

### 4.4 Temporal correspondence between swelling and maximum mucoadhesion

A particularly relevant observation in the present study was the close temporal correspondence between the experimentally observed maximum swelling and the estimated maximum adhesion tension. Both IDB-MS and DFP-MS reached their maximum experimentally observed swelling at 5 min, whereas maximum adhesion occurred at approximately 4.0–4.5 min depending on formulation and hydration condition. The estimated adhesion maxima therefore occurred approximately 0.5–1.0 min before the experimentally observed swelling maximum. The ascending phases provide additional information. Under native/quasi-dry conditions, the ascending adhesion profile showed closer temporal correspondence with the swelling profile, whereas under highly hydrated conditions adhesion developed substantially faster than bulk swelling. At 75% of the maximum response, the difference was approximately 2 min under highly hydrated conditions (1.1–1.2 vs. 3.4–3.5 min). This temporal correspondence is consistent with models in which hydration and swelling contribute to the progressive establishment of the adhesive interface. Studies of developing bioadhesive bonds have demonstrated that water movement within the interface is spatially associated with changes in mucin distribution (Ugwoke et al., 2001; Smart, 2005; Khutoryanskiy, 2011). Using NMR microscopy and confocal microscopy in an alginate–mucin model, Marshall et al. (2004) demonstrated characteristic water-diffusion and mucin-concentration profiles within the developing adhesive bond, supporting the concept that hydration and polymer–mucin redistribution are integral components of interface formation.

Because swelling and mucoadhesion were measured in separate experiments, the observed temporal correspondence should not be interpreted as evidence of direct causal coupling. Swelling represents only one of several determinants of mucoadhesion, alongside polymer chemistry, chain flexibility, molecular weight, crosslinking, functional-group availability, mucus composition, and contact conditions.

In addition, the hydration environments in the two assays were not equivalent. Gravimetric swelling was determined after immersion of the compacted discs in bulk PBS, whereas mucoadhesion developed at the tissue interface under restricted and hydration-dependent fluid availability. Accordingly, the swelling profile represents the bulk hydration capacity of the standardized compacted samples and not the local hydration state of the polymer at the mucosal interface. The comparison between the two profiles should therefore be interpreted as a temporal association between independently measured processes rather than as evidence that identical hydration kinetics occur under the two experimental conditions.

### 4.5 Different swelling–adhesion relationships under highly hydrated and native mucosa

Comparison of the normalized kinetic profiles revealed an important difference between the two mucosal conditions. Under native/quasi-dry conditions, the ascending phase of adhesion showed relatively close temporal correspondence with swelling. In contrast, under highly hydrated conditions, adhesion developed considerably faster than the bulk swelling response: 75% of maximum adhesion was reached after approximately 1.1–1.2 min, whereas 75% of maximum swelling required approximately 3.4–3.5 min. This observation suggests that development of substantial bulk swelling is not required for the initial establishment of measurable mucoadhesion. Surface hydration and polymer-chain mobilization at the polymer–mucus interface may occur before the entire polymer matrix reaches the corresponding degree of gravimetrically measured swelling. This interpretation is compatible with the established contact–consolidation model, in which initial wetting and local hydration precede further polymer–mucin interpenetration and consolidation. More importantly, rapid establishment of adhesion under highly hydrated conditions did not result in greater adhesive strength. Instead, absolute adhesion tension was substantially lower under highly hydrated conditions. These findings indicate that the rate of adhesion development and the mechanical strength of the resulting adhesive interface represent distinct aspects of mucoadhesive performance. One possible interpretation is that the highly hydrated environment provides more rapid access of water to the particle surface, which may facilitate earlier mobilization of surface polymer chains. However, the presence of a larger aqueous phase may reduce the effective polymer concentration within the interfacial region and thereby decrease the cohesive strength of the hydrated polymer layer. These processes were not directly measured in the present study and should therefore be regarded as plausible mechanistic interpretations rather than experimentally demonstrated mechanisms. The literature supports this general mechanism: excessive hydration and swelling can facilitate removal of a mucoadhesive layer from the mucosal surface, while over-dilution of polymer chains can weaken the adhesive bond (Marshall et al., 2004; Popeski-Dimovski, 2015). The present results are therefore consistent with a distinction between rapid hydration-associated development of adhesion and formation of a mechanically stronger, more persistent mucoadhesive interface.

### 4.6 Post-maximum behavior: swelling and adhesion diverge

The behavior after the maximum provides further evidence that swelling alone cannot account for the measured mucoadhesive strength. After 5 min, the swelling index remained close to its maximum at 10 min and subsequently decreased gradually. Even after 40 min, IDB-MS and DFP-MS retained approximately 81% and 79% of their respective maximum swelling indices. In contrast, adhesion decreased substantially earlier: it fell to 75% of maximum at approximately 7.2 min under highly hydrated conditions and at approximately 10.9–11.0 min under native/quasi-dry conditions. Thus, the decline in mucoadhesive strength clearly preceded a comparable decrease in bulk swelling. The post-maximum decrease in adhesion tension should not be attributed to a single mechanism on the basis of the present data. Several processes may contribute, including progressive polymer hydration and relaxation, reduced cohesive strength of the hydrated polymer layer, accumulation of interfacial water with a possible lubricating effect, and time-dependent changes at the mucus–polymer interface. The present experimental design does not allow the relative contribution of these processes to be distinguished. Therefore, the decrease in adhesion observed after approximately 5 min is interpreted here as an experimentally established kinetic feature, whereas the mechanisms proposed to explain it remain hypotheses requiring direct experimental verification. This finding argues against a simple model in which adhesion tension is directly proportional to the gravimetrically determined water uptake of the compacted microsphere samples. Instead, once sufficient hydration has been achieved, other properties of the interface appear to become increasingly important. A decrease in gravimetrically determined swelling after the maximum may reflect several overlapping processes, including polymer-chain relaxation and rearrangement, partial dissolution or erosion, changes in water retention, or mechanical loss of material during handling. Similar post-maximum decreases have been reported experimentally for alginate-containing particulate systems. For example, alginate/gelatin microparticles showed a swelling maximum followed by a gradual decrease that the authors associated with polymer dissolution (Szekalska et al., 2023b). Other alginate-based microbead studies have reported swelling followed by erosion or dissolution of the hydrated matrix (Nayak et al., 2011; Khan et al., 2022). However, the present gravimetric method does not allow these mechanisms to be distinguished. Therefore, the observed decrease should be described as a post-maximum decrease in apparent swelling rather than as direct evidence of polymer erosion or dissolution. Consequently, the gravimetric data alone cannot determine whether the post-maximum change reflects reduced water retention by the remaining polymer, loss of polymeric material, or a combination of both processes. Accordingly, the temporal comparison with mucoadhesion is based on the experimentally determined gravimetric swelling index and should not be interpreted as evidence that changes in polymer swelling alone caused the corresponding changes in adhesion.

### 4.7 Proposed hydration-dependent model of mucoadhesion

Taken together, the findings indicate that the relationship between polymer hydration and mucoadhesion is not adequately described by a simple positive relationship between water uptake and adhesive strength. Under native/quasi-dry conditions, adhesion developed more gradually and reached substantially higher values, whereas under highly hydrated conditions adhesion developed more rapidly but remained weaker and declined earlier.

The present study compared two distinct mucosal hydration conditions and was therefore not designed to experimentally determine an optimal hydration level. Nevertheless, the higher adhesion observed under the native/quasi-dry condition compared with the highly hydrated condition, together with the time-dependent relationship between swelling and adhesion, is consistent with the hypothesis that mucoadhesion may depend on a balance between sufficient polymer hydration and preservation of interfacial cohesion. Establishing whether an intermediate or optimal hydration range exists would require systematic evaluation across multiple quantitatively defined levels of mucosal hydration.

It should be emphasized that polymer-chain mobility, interfacial hydration, polymer–mucin interpenetration, and changes in cohesive properties were not directly measured in the present study. Accordingly, the mechanisms discussed above should be regarded as plausible interpretations consistent with the observed temporal relationships between swelling, mucosal hydration, and mucoadhesion, rather than as experimentally demonstrated mechanisms. Direct verification of these mechanisms would require complementary physicochemical and structural analyses.

### 4.8 Relevance to nasal drug delivery

The observed hydration dependence has implications for the experimental design and evaluation of nasal mucoadhesive formulations. Nasal delivery systems must interact with a continuously hydrated mucus layer while simultaneously resisting mucociliary clearance. Increasing residence time at the nasal mucosa is one of the principal reasons for incorporating mucoadhesive polymers into nasal delivery systems (Illum, 2003). The literature on nasal mucoadhesive systems emphasizes that polymer hydration must reach an appropriate level: insufficient hydration restricts chain mobility, whereas excessive hydration may weaken polymer–mucus interactions and facilitate subsequent clearance (Andrews et al., 2009; Frent et al., 2022). Previous studies have demonstrated the potential of alginate-based microspheres as nasal mucoadhesive carriers, including evidence of prolonged nasal retention (Tafaghodi et al., 2004; Patil and Sawant, 2009; Patil et al., 2012). The present findings indicate that mucoadhesive performance cannot be explained by the intrinsic swelling capacity of the polymeric carrier alone. Although IDB-MS and DFP-MS exhibited highly similar swelling kinetics, the strength and temporal persistence of their mucoadhesive interaction with nasal mucosa depended strongly on the hydration state of the biological interface. This has methodological implications as well. *Ex vivo* mucoadhesion measurements performed exclusively on extensively hydrated mucosal tissue may characterize a different interfacial state from that occurring under native/quasi-dry or less extensively hydrated conditions. Since mucus secretion, turnover, moisture content and other physiological characteristics influence mucoadhesion, these parameters should be considered when designing and interpreting experimental models of nasal drug delivery (Frent et al., 2022; Szekalska et al., 2018). Accordingly, evaluation of polymeric nasal carriers under more than one hydration condition may provide a more informative assessment of their mucoadhesive behavior than testing under a single highly hydrated condition.

From a formulation perspective, the objective should therefore not necessarily be to maximize polymer swelling, but rather to achieve a controlled hydration profile that provides sufficient chain mobility while preserving interfacial cohesion and prolonged attachment to the nasal mucosa.

Several methodological limitations should be considered when interpreting the present findings. First, the experiments were performed using excised ovine nasal mucosa, and tissue viability, mucin-layer integrity, and possible time-dependent autolytic changes were not independently quantified during the experimental period. Although freshly isolated tissue was used within a controlled time frame and handled consistently across experimental groups, potential changes in the *ex vivo* mucosal surface during prolonged contact cannot be completely excluded.

Second, both swelling and mucoadhesion were evaluated using standardized compacted microsphere discs rather than freely dispersed microspheres. Compaction was necessary to obtain reproducible sample geometry and a defined contact area for quantitative force measurements, but it may modify surface area, porosity, water penetration, and polymer relaxation compared with the uncompacted microsphere formulation. Consequently, the present results characterize the hydration and mucoadhesive behavior of the formulations under the standardized experimental configuration used and should not be interpreted as a direct simulation of freely dispersed microspheres following nasal administration.

## 5 Conclusion

Under the standardized experimental conditions used in this study,the mucoadhesive behavior of the investigated polymeric nanocomposite microsphere systems was strongly influenced by the hydration state of the ovine nasal mucosa. IDB-MS and DFP-MS exhibited highly similar swelling kinetics and no significant formulation-dependent difference in adhesion tension, whereas adhesion strength was substantially higher under native/quasi-dry than under highly hydrated mucosal conditions. Both systems reached their highest experimentally observed swelling at 5 min, temporally close to the spline-estimated maximum adhesion at approximately 4.0–4.5 min. However, the hydration-dependent differences in the ascending and descending phases indicate that gravimetric swelling kinetics alone are insufficient to explain mucoadhesive strength and persistence. The findings support the hypothesis that effective mucoadhesion may depend on a balance between sufficient polymer hydration and preservation of interfacial cohesion; however, the present comparison of two hydration conditions does not establish an optimal hydration level. These results highlight mucosal hydration as an important experimental consideration when evaluating polymeric mucoadhesive systems for nasal drug delivery.

## Data Availability

The raw data supporting the conclusions of this article will be made available by the authors, without undue reservation.
